# Effects of wearable visual cueing on gait pattern and stability in patients with Parkinson’s disease

**DOI:** 10.3389/fneur.2023.1077871

**Published:** 2023-03-24

**Authors:** Wei Zhang, Yun Han, Yuanyuan Shi, Shilei Yan, Wenjing Song, Guiyun Cui, Jie Xiang

**Affiliations:** ^1^Jiangsu Key Laboratory of Brain Disease Bioinformation, Xuzhou Medical University, Xuzhou, Jiangsu, China; ^2^Department of Neurology, The Affiliated Hospital of Xuzhou Medical University, Xuzhou, Jiangsu, China; ^3^Department of Neurology, Suining County People’s Hospital, Xuzhou, Jiangsu, China; ^4^Department of Rehabilitation, The Affiliated Hospital of Xuzhou Medical University, Xuzhou, Jiangsu, China; ^5^The First Clinical Medicine College, Xuzhou Medical University, Xuzhou, Jiangsu, China

**Keywords:** Parkinson ‘s disease, gait pattern, stability, neurorehabilitation, wearable visual cueing

## Abstract

The present study examined the effects of wearable visual cues, provided by a wearable laser device, on the gait pattern and stability in patients with Parkinson’s disease (PD). In total, 18 patients with a clinical diagnosis of idiopathic PD (Hoehn and Yahr stage II-III) and 18 healthy controls were included. The main outcome measures included spatiotemporal parameters, sagittal plane kinematic parameters of joints in lower limbs, and dynamic center of pressure (COP) parameters. Significant intra-group improvement in gait parameters was observed in PD patients. Compared with that at baseline, the gait pattern improved in PD patients under the cued condition, with longer stride length and higher toe clearance, as well as shortening of double stance phase, especially the stride length, double stance phase and toe clearance were not significantly different between cued condition and healthy control groups. In kinematics, the ankle peak dorsiflexion in swing phase and the hip range of motion (ROM) in gait cycle was significantly improved in PD patients with visual cues and close to healthy controls. Decreased anteroposterior (AP) position of COP improved gait stability in patients with PD under the cued condition. Multiple linear regression analysis showed that the AP position has a negative correlation with ankle peak dorsiflexion in swing phase. Pearson’s correlation coefficients showed that the minimum toe clearance (Mini TC) was positively correlated with the ankle peak dorsiflexion in swing phase. The immediate effect of wearable visual cues improved the gait pattern and stability in PD patients, suggesting that it may be effective when applied as an alternative technique in rehabilitation training for PD patients.

## Introduction

Parkinson’s disease (PD) affects 0.3% of the global population, and approximately 1% of people aged over 60 years (1). It is the second most common neurodegenerative disease worldwide, with its incidence and prevalence increasing due to changing population demographics. PD is caused by a deficiency in the neurotransmitter dopamine in the basal ganglia region of the brain. This evokes characteristic changes in motor activity, which include resting tremor, rigidity, postural instability, and bradykinesia (2). These changes translate into various physical impairments, including a particularly destructive effect on gait.

An abnormal gait pattern, involving freezing of gait (FOG) or a festinating gait, is one of the leading causes of disability in patients with PD (3, 4). Gait deficits, including reduced stride length and gait instability, gradually worsen with disease progression, often resulting in accidental falls and lost locomotion ability in patients, eventually leading to medical and social problems (5, 6).

Dopaminergic drugs are included in the therapies aiming to correct motor disturbances (7), and surgical therapy involving deep brain electrical stimulation has become a successful symptomatic treatment strategy for PD (8). However, gait deficits with disease progression are highly common in PD patients (9). Studies have indicated that although PD patients exhibit an abnormal gait pattern, they probably experience problems in the activation of the motor control system regulated by cortical-striatal loop (10, 11). It has been suggested that in PD, basal ganglia dysfunction may cause gait and postural problems due to a progressive reduction in dopaminergic neurons in the substantia nigra (12). PD patients may use visual cueing to provide spatial information to guide movements, which may allow them to bypass their defective basal ganglia during walking. Therefore, assessing the efficacy of alternative approaches for the management of gait deficits is essential.

The use of visual cues to improve freezing of gait was reported as early as 1990 (13), which was considered to have a direct and significant biomechanical effect on the gait performance in patients with PD experiencing gait deficits (9, 14). In 2010, Espay et al. observed a positive effect of visual cueing on the mean FOGQ score and a trend toward an improvement in the frequency of FoG (15). In the same year, Bryant and colleagues presented a walking cane device that projected a continuous visual cueing *via* an attached laser and reported a significant positive immediate effect of a green laser line on the mean frequency of FOG episodes (16). In 2016, research attention shifted to the development of visual cueing systems that were more wearable (17). A mobile line provided by laser, so as a fixed transverse line on the floor, has been shown to be associated with improved gait patterns, such as increased stride length (18, 19), velocity (18, 20), push-off force (18), and normalized angles for kinematic parameters (11, 21) in PD patients. Therefore, wearable visual cueing methods have become an important research interest.

Till now, multiple studies have investigated the effects of wearable visual cuing on PD gait. Zhao et al. demonstrated that Google Glass and a walker with a built-in laser can improve gait performance with normalized stride length and cadence, thereby causing a statistically significant reduction in freezing of gait questionnaire (FOGQ) scores and the frequency of falls in PD patients with freezing of gait (FOG) (22). Recently, Barthel et al. found that the immediate efficacy of laser shoes offer a promising intervention with potential to deliver in-home cueing for patients with FOG (23). However, such wearable devices are lacking in ease application during daily life. Most importantly, these studies did not evaluate the effect of visual cues on postural stability.

Postural instability is possibly the most challenging of the major PD movement symptoms, as it is associated with an increased risk of falls and subsequent medical complications(e.g., fractures) (24). Over the past 30 years, the center of pressure (COP) has been commonly used for evaluating postural control, however examined during locomotion (dynamic COP) are rarely studied. A recent study has discovered that the overlaid graphical display of dynamic COP trajectory showed “distorted butterfly with asymmetric wing” feature in PD patients (25). The COP measures included the following parameters: the length of gait line (LGL), the length of single support line (SSL), anterior/posterior (AP) position and lateral symmetry. The present study aimed to investigate the effects of visual cues produced by a wearable laser device on the gait pattern and address the effect of cueing on postural stability using COP measurements in PD patients during walking for the first time in a controlled environment. The findings of this study may provide more information on the application of visual cues in PD. The operation of a wearable laser device is simple, thus, patients can easily use this device at home. We hypothesize that the dynamic COP trajectories can be improved and that they can walk stably with the aid of a wearable visual cue device.

## Materials and methods

### Subjects

For this study, 18 patients with PD were recruited from the Affiliated Hospital of Xuzhou Medical University and 18 healthy controls (HC) were recruited from local communities. The age, weight, and height in the HC group were comparable to patients with PD. The patients’ inclusion criteria were: (i) diagnosed with idiopathic PD according to the UK Brain Bank Clinical Criteria; (ii) Hoehn-Yahr (H&Y) stage II or III; (iii) able to walk without any physical assistance or ambulatory aids. The exclusion criteria were: (i) comorbidities of neurological disease other than PD or musculoskeletal problems affecting walkability and postural stability; (ii) Impaired cognition [Mini-Mental State Examination (MMSE) ≤ 24]; (iii) inadequate vision to perceive the cues; (iv) exhibited freezing of gait during the “on” stage of the medication for PD that affected the quality of COP data. The following test on the modified Unified Parkinson’s Disease Rating Scale (UPDRS) was conducted by two trained neurologists. Details of the PD and HC groups are shown in Table 1.

**Table 1:** Demographic characteristics of PD and HC groups.

	PD (*n* = 18)	HC (*n* = 18)
Sex, M/F	12/6	10/8
Age (years)	63.06 ± 8.63	65.89 ± 6.09
Height (m)	1.64 ± 0.07	1.59 ± 0.06
Weight (kg)	71.58 ± 10.81	64.18 ± 13.24
BMI (kg/m^2^)	26.53 ± 3.28	25.26 ± 3.67
MMSE	26.94 ± 2.21	**29.51 ± 0.71**
TUG (s)	10.74 ± 2.2	**6.88 ± 1.13**
MORSE	28.05 ± 21.29	**5.55 ± 7.04**
H&Y	2.55 ± 0.44	
UPDRS III	31.27 ± 11.44	
Disease Duration(years)	8.77 ± 2.1	

M, male; F, female; BMI, body mass index; MMSE, mini-mental state examination; TUG, timed up and go test; MORSE, morse fall scale score; H&Y, Hoehn & Yahr; UPDRS III, unified Parkinson’s disease rating scale part III. Bold values denote statistical significance at the *p* < 0.05 level.

This study was approved by the Ethics Committee of the Affiliated Hospital of Xuzhou Medical University (XYFY2020-KL105-01). All participants were reviewed and signed an informed consent document before participation.

### Experimental setup and procedures

**Figure 1:**
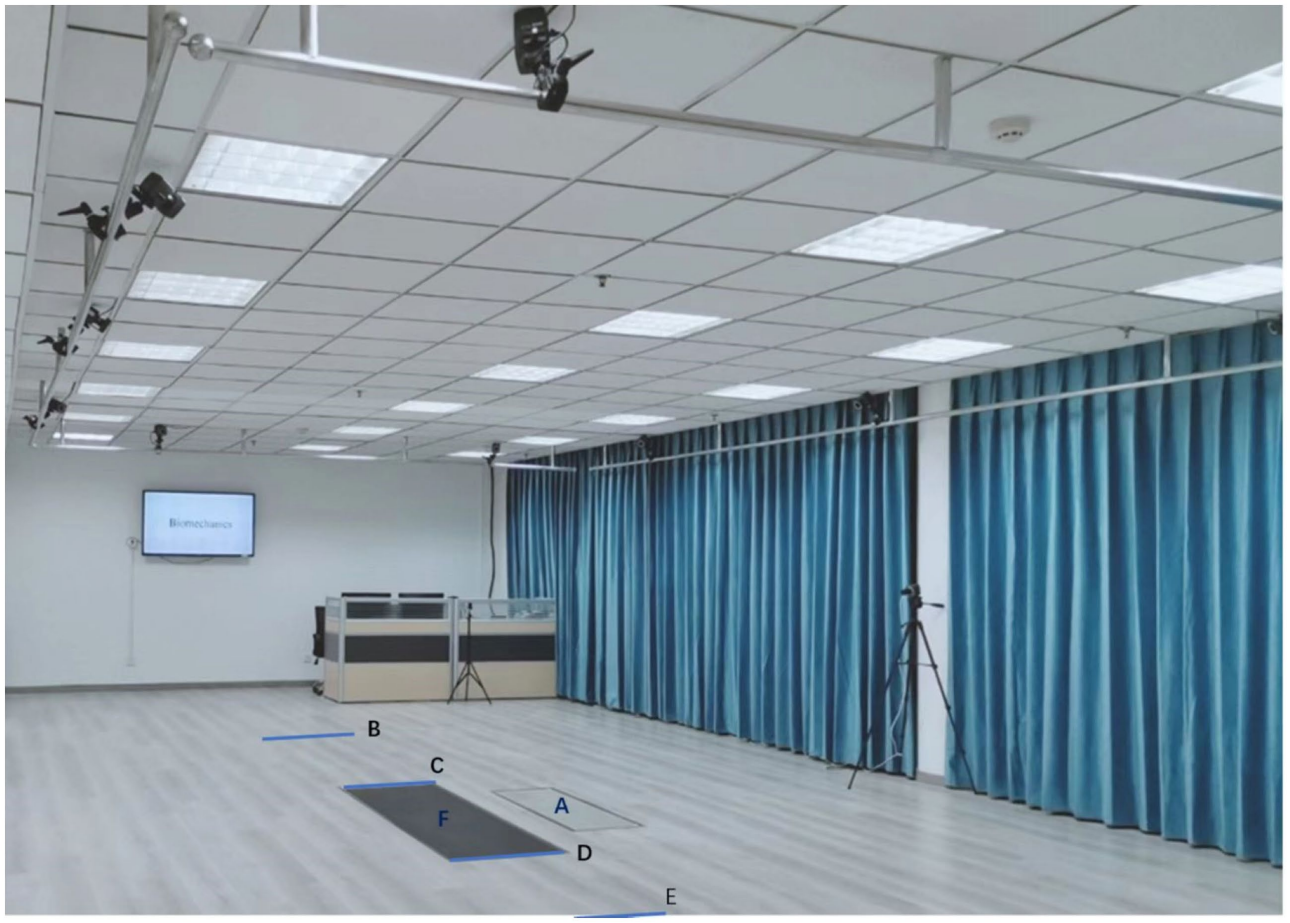
Experimental setup. (A) Plantar pressure plate A. The distance between line (B) and (C), line (C) and (D), line (D) and (E) is 2 meters. (F) Plantar pressure plate F.

All gait tests were conducted at the Biomechanics and Motion Analysis Laboratory of Xuzhou Medical University (Figure 1), and all PD clinical assessments were conducted in the practically “ON” state. The laboratory space was surrounded by a three-dimensional motion capture system with 10 infrared video cameras (Vicon Motion Systems Ltd., Oxford, UK). A plantar pressure plate (FDM2, Zebris Medical GmbH, Germany) was mounted under the walkway. The capture system and plantar pressure plate were both sampled at 100 Hz.

**Figure 2:**
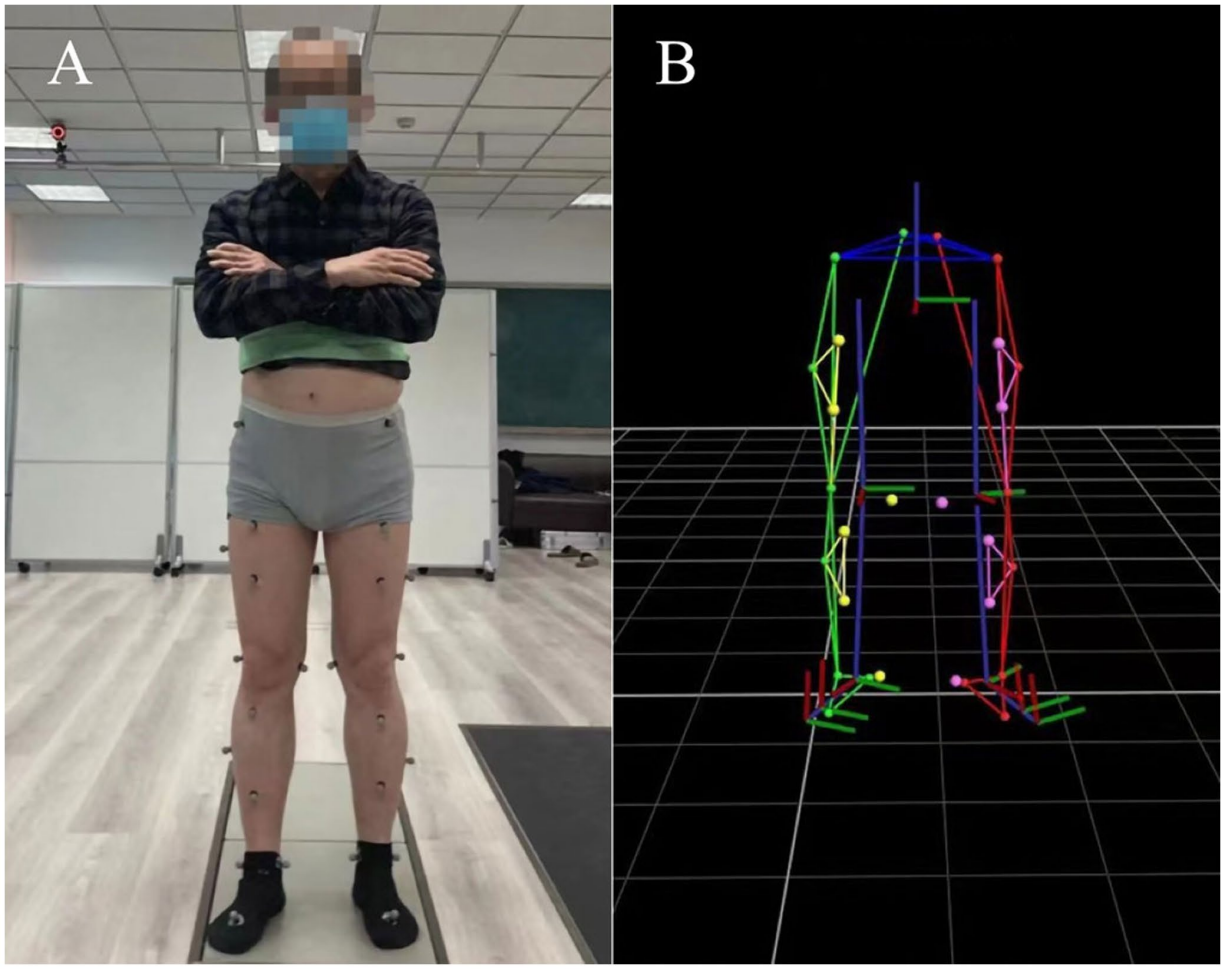
Front view of the subject equipped with reflective markers and the static lower limb model.

In this experiment, 28 reflective markers (diameter:14 mm) of a modified Cleveland Clinic marker set were attached with double-sided adhesive tape to the anatomical landmarks according to the plug-in gait lower limb 2.3 model (26, 27) (Figure 2). Different from previous marker set, we added two reflective marker locations included right and left posterior superior iliac spine in order to better calculate hip joint angles. Before the gait test measurements, all subjects were asked to walk around the laboratory to familiarize themselves with the experimental procedure and environment at their preferred speed. A static calibration was firstly carried out for 3 s when the participant stood on the plantar pressure plate A (Figure 1) to generate a Model Coordinate System of lower limb in a neutral position(Figure 2B).

**Figure 3:**
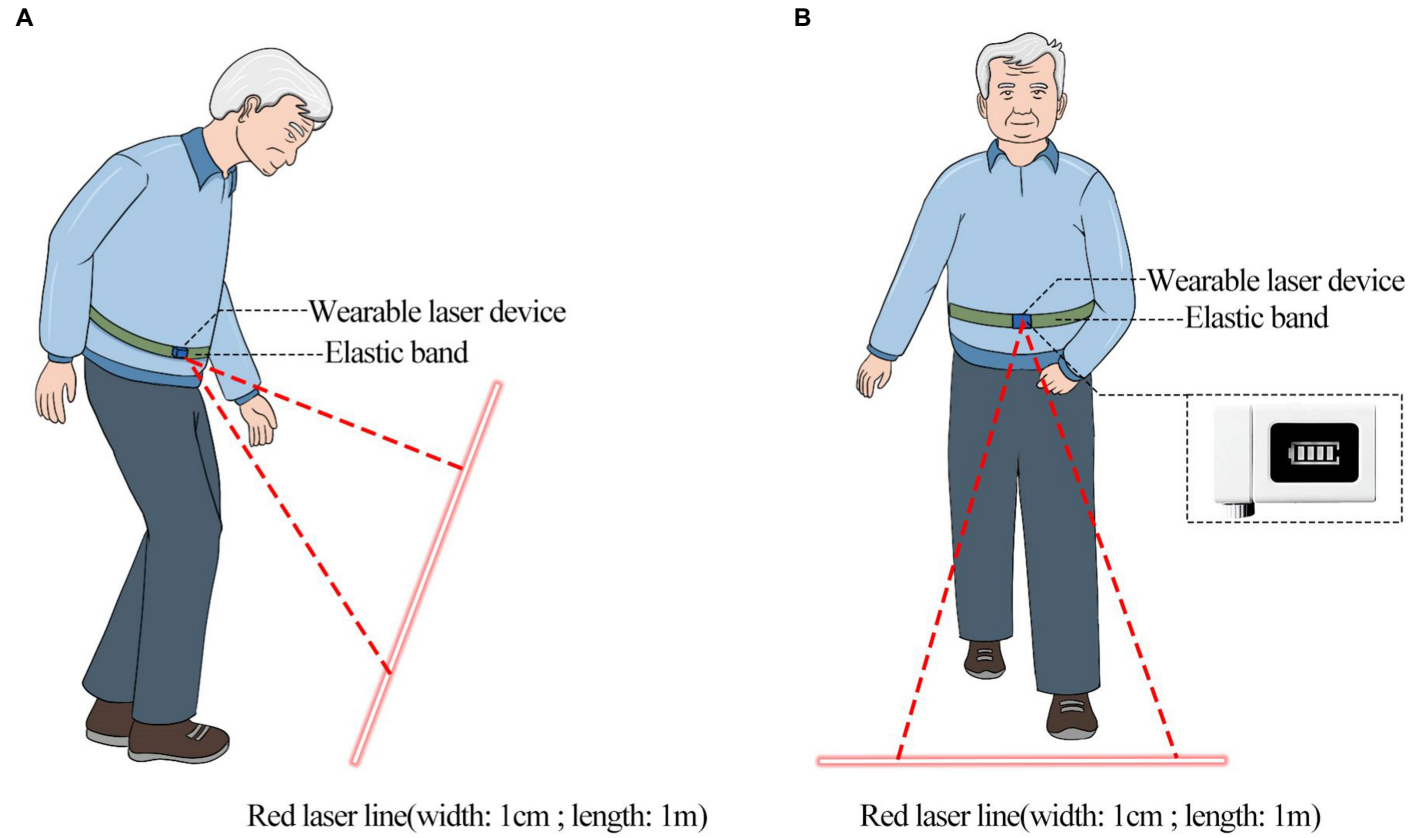
Description of the cued condition. **(A)** Side view of the PD patient in cued condition; **(B)** front view of the PD patient in cued condition.

We collected gait data of the PD group under two conditions: no visual cues first (baseline) and then with visual cues (cued condition). In the cued condition (Figure 3), the visual cues were provided by a wearable laser device, which consisted of a lithium battery, a universal bracket, and an infrared light-emitting diode. Since this study aimed to access if visual cues can normalize the gait pattern and postural stability of PD patients, making them close to the HC group, the latter was not assessed in the cued condition. The wearable laser device with adjustable beam angle was held in place on the participant’s waist with an elastic band, and projected a 1 cm wide, red transverse laser line onto the ground ahead of the participant. The individual was instructed to touch their toes to the laser line and to continue to walk, repeating this procedure in sequence with the movement of the laser. The projection was approximately 110% of the average step length in front of the patients’ toes (28).

The subjects were instructed to walk straight at a self-selected speed along the 6 m walkway(from line B to line E in Figure 1) back and forth in order to guarantee that 5 gait cycles were recorded over the plantar pressure plate F (Figure 1) for PD patients in two conditions and the HC group. The marker trajectories were post-processed with a fourth-order low-pass filter at 6 Hz. The gait tests in the PD group were performed during the on-phase of the medication cycle.

### Variables and analysis

The spatiotemporal, kinematic, and dynamic COP variables were of interest in the present study. The spatiotemporal variables included stride length, cadence, gait velocity, stance and double stance phases, toe clearance (TC). Toe height at minimum toe clearance (Mini TC) was computed as the toe vertical local minimum between the first maximum (Max TC1) following toe-off and the second maximum (Max TC2) of vertical displacement (29). Kinematic variables included the motion of the ankle, knee, and hip joints and pelvis in the sagittal plane. COP variables included length of gait line (LGL) and single limb support line (SSL), AP and lateral symmetry.

A total of 5 gait cycles including left and right were collected and averaged for each subject. The gait cycle was identified as the period of time from the initial contact of one foot to the following occurrence of the same event with the same foot. The spatiotemporal and kinematic variables were pre-processed using Nexus software (version 2.6, Vicon, Oxford, UK), by manually defining gait cycle events, then were post-processed by Visual 3D software (C-Motion Inc., Germantown, MD). Kinematic profiles were time-normalized to 101 data points, representing the percentage of the gait cycle, which was specified as the time between two sequential foot strikes, and they were averaged for the 5 gait cycles of both lower limbs. The COP variables were processed using Zebris FDM 1.18 software (Zebris Medical GmbH, Isny, Germany), and a gait analysis report was created which included COP butterfly and gait line information. The LGL and SSL were normalized by dividing by each subject’s foot length to eliminate the influence of different sized feet. The variables of AP position and lateral symmetry were taken as absolute values.

Statistical analyses were performed using the SPSS statistical software package 20.0 (IBM, corporation, Armonk, NY, USA). Demographic data were presented as mean ± SD. The normality of the distribution of each variable was tested using the Shapiro–Wilk test. Homogeneity of variance is assessed using Levene’s Test for equality of variances. We conducted One-way ANOVA on spatiotemporal, kinematic and COP variables. When the assumption of homogeneity of variances is satisfied, Bonferroni correction is adopted for multiple pairwise comparisons, otherwise the Games-howell test is used. Multiple regression analysis was used to analyze the linear relationship between a dependent variable and multiple independent variables. Bivariate analysis was performed to explore the relationship between two variables. *p* < 0.05 was considered statistically significant.

## Results

### Spatiotemporal variables

Table 2 summarizes the spatiotemporal data of the PD and HC groups. Compared with the HC group, the PD group in baseline showed significantly lower stride length (*p* < 0.001) and gait velocity (*p* < 0.001), as well as prolonged stance and double stance phase of gait cycles (*p =* 0.009 and *p =* 0.002) and reduced Max TC2 and Mini TC (*p* < 0.001). Additionally, these spatiotemporal data revealed the significant main effect of the condition in comparison between the PD group in baseline and the cued condition. When compared with the PD baseline group, the results in the cued condition indicated a higher value on stride length (*p =* 0.013), a lower value on double stance phase (*p =* 0.036), a higher value on Max TC2 and Mini TC (*p =* 0.038 and *p =* 0.024). Particularly, the value of stride length, double stance phase, Max TC2 and Mini TC in the cued condition were comparable to the HC group (*p* > 0.05).

**Table 2:** Spatiotemporal variables of PD and HC groups.

Variables	HC	PD-I	PD-II	PD-I vs. HC	PD-II vs. HC	PD-I vs. PD-II.
	Mean ± SD	Mean ± SD	Mean ± SD	Value of *p*	Value of *p*	Value of *p*
Stride length (m)	1.20 ± 0.12	1.02 ± 0.13	1.14 ± 0.12	**0.000**	0.317	**0.013**
Cadence (steps/min)	119.34 ± 8.75	113.94 ± 11.10	108.84 ± 7.69	0.262	**0.004**	0.317
Velocity (m/s)	1.20 ± 0.16	0.96 ± 0.10	1.03 ± 0.10	**0.000**	**0.001**	0.225
Stance phase (%GC)	63.07 ± 0.83	64.36 ± 1.44	63.59 ± 1.35	**0.009**	0.632	0.210
Double stance phase(%GC)	26.14 ± 1.81	28.89 ± 2.61	26.90 ± 2.36	**0.002**	0.956	**0.036**
Max TC1 (mm)	85.41 ± 6.92	90.33 ± 13.79	93.70 ± 18.91	0.381	0.211	0.815
Max TC2 (mm)	108.63 ± 12.22	87.02 ± 18.37	100.76 ± 16.61	**0.000**	0.435	**0.038**
Mini TC (mm)	63.33 ± 5.93	50.46 ± 11.13	59.30 ± 10.84	**0.001**	0.641	**0.024**

PD-I, PD baseline group; PD-II, PD cued condition group; HC, healthy control group. TC, toe clearance. Bold values denote statistical significance at the *p* < 0.05 level.

### Kinematic variables

**Figure 4:**
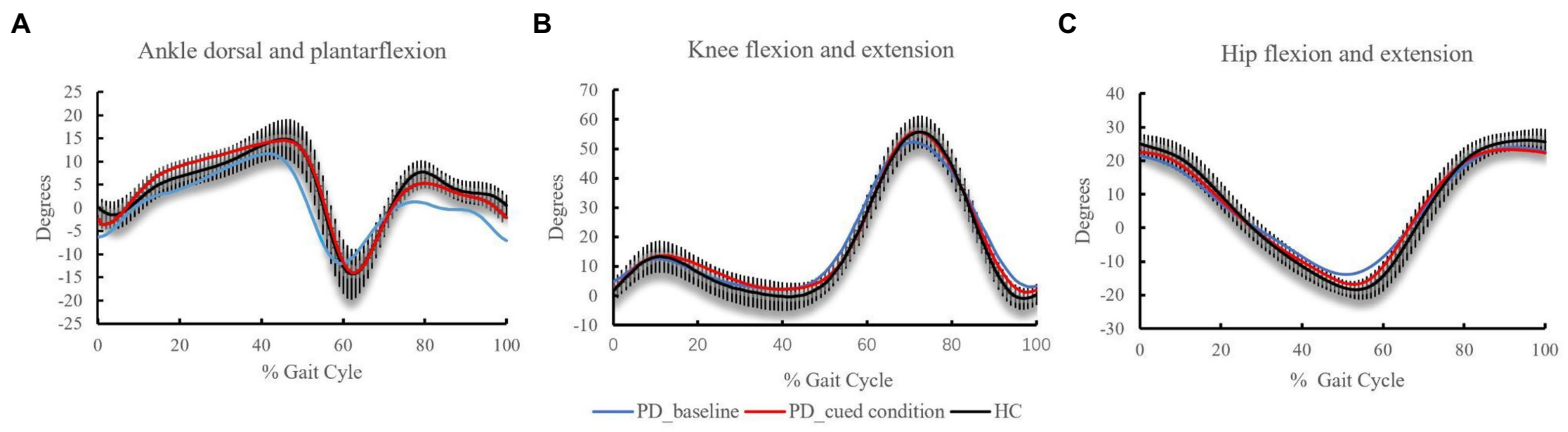
Kinematic data at the **(A)** ankle, **(B)** knee, and **(C)** hip across the gait cycle. Positive angles indicate joint flexion and dorsiflexion; negative angles represent extension and plantarflexion.

Pronounced differences were observed in the kinematic data and profiles between the PD group at baseline and healthy control group (Table 3; Figure 4). The angle at initial contact of the ankle joints in PD group at baseline, was increased compared with healthy control group (*p* = 0.010), while the peak dorsiflexion in swing phase and ROM in swing phase was decreased (*p* = 0.015 and 0.043). Particularly, the peak dorsiflexion in swing phase change significantly with the visual cueing intervention (*p* = 0.040) show non-significant difference with HC group (*p =* 0.802).

**Table 3:** Kinematic variables of PD and HC groups.

Variables	HC	PD-I	PD-II	PD-I vs. HC	PD-II vs. HC	PD-I vs. PD-II.
	Mean ± SD	Mean ± SD	Mean ± SD	Value of *p*	Value of *p*	Value of *p*
*Ankle(degree)*						
Angle at initial contact	−2.02 ± 2.91	−5.8 ± 3.96	−5.04 ± 4.01	**0.010**	**0.051**	1.000
Peak dorsiflexion in stance phase	14.14 ± 4.01	12.21 ± 7.07	14.15 ± 6.94	1.000	1.000	1.000
Plantarflexion at toe off	−12.6 ± 5.46	−10.66 ± 6.99	−9.97 ± 6.62	1.000	0.671	1.000
Peak dorsiflexion in swing phase	5.27 ± 2.81	1.63 ± 4.33	4.70 ± 2.60	**0.015**	0.802	**0.040**
Peak plantarflexion in swing phase	−13.21 ± 5.42	−12.65 ± 6.26	−11.98 ± 6.05	1.000	1.000	1.000
ROM in swing phase	18.48 ± 5.3	14.69 ± 4.02	15.55 ± 3.98	**0.043**	0.167	1.000
ROM in gait cycle	27.63 ± 4.34	25.15 ± 4.76	26.44 ± 5.16	0.378	1.000	1.000
*Knee(degree)*						
Angle at initial contact	2.99 ± 1.61	4.42 ± 4.19	2.97 ± 1.60	0.377	1.000	0.374
Peak flexion in stance phase	15.56 ± 4.19	13.48 ± 5.11	14.48 ± 7.76	0.388	0.863	0.892
Peak extension in stance phase	1.68 ± 4.58	0.68 ± 3.77	0.55 ± 4.35	1.000	1.000	1.000
Flexion at toe off	37.95 ± 8.01	37.05 ± 7.16	35.88 ± 6.52	1.000	1.000	1.000
Peak flexion in swing phase	56.17 ± 5.3	54.04 ± 6.63	53.56 ± 6.41	0.910	0.629	1.000
ROM in swing phase	54.90 ± 4.21	52.9 ± 6.57	54.32 ± 5.91	0.883	1.000	1.000
ROM in gait cycle	55.55 ± 3.39	53.99 ± 6.17	54.71 ± 5.71	1.000	1.000	1.000
*Hip(degree)*						
Flexion at initial contact	25.73 ± 5.12	21.29 ± 6.15	22.70 ± 7.04	0.104	0.435	1.000
Angle at toe-off	−6.49 ± 7.00	−4.36 ± 6.24	−6.34 ± 7.00	1.000	1.000	1.000
Peak flexion in swing phase	27.64 ± 5.23	24.14 ± 5.64	24.76 ± 6.42	0.227	0.429	1.000
Peak hip extension in gait circle	−16.56 ± 6.45	−14.78 ± 5.5	−16.38 ± 6.52	1.000	1.000	1.000
ROM in gait cycle	44.41 ± 3.66	38.61 ± 4.72	42.43 ± 4.76	**0.001**	0.556	**0.036**

PD-I, PD baseline group; PD-II, PD cued condition group; HC, healthy control group; ROM, range of motion. Bold values denote statistical significance at the *p* < 0.05 level.

Regarding the knee joint, the angles showed no significant difference between three groups (*p* > 0.05). For the hip joint, when compared with the HC group, PD patients at baseline showed reduced ROM in gait cycles (*p* = 0.001), which was found to increase significantly with the interaction of visual cues (*p* = 0.036).

### COP variables

**Figure 5:**
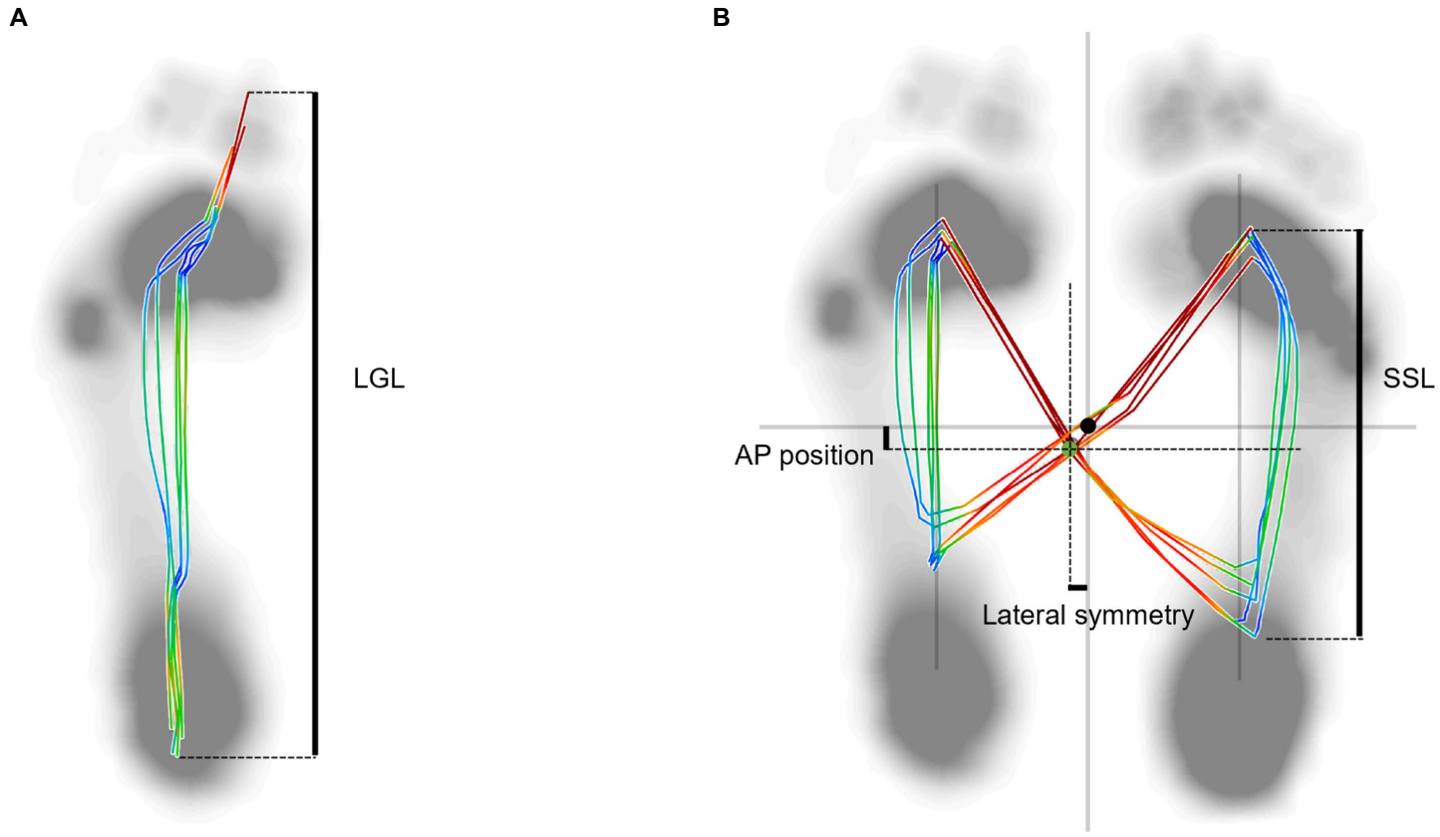
Plantar center of pressure trajectory variables of a 59-year-old female PD patient with left-side dominant motor symptoms. **(A)** Length of gait line (LGL): the length of trajectory generated from one side of the heel lands on the ground to the toes off. This parameter covers the progression of the COP of all the steps recorded of one side of the body. **(B)** Butterfly diagram. AP position: This parameter describes the shift forwards or backwards of the COP intersection point in chronological sequence in the cyclogram display, taking all the steps into consideration. SSL: the length of the movement of the COP during single limb support. Lateral symmetry: This parameter is an absolute value, which describes the left/right shift of the COP intersection point in chronological sequence in the cyclogram display, taking all the steps into consideration. The black dot point indicates the zero position, and the green dot point indicates the intersection point of COP trajectory.

The PD patients in baseline showed lower values in SSL and larger AP position and lateral symmetry compared with HC group (*p =* 0.008, 0.000, and *p =* 0.045) (Table 4; Figure 5). The result of the AP position in PD patients in the cued condition showed a significantly smaller value than the baseline (*p =* 0.018) and was comparable to the HC group (*p =* 0.692).

**Table 4:** COP variables of PD and HC groups.

Variables	HC	PD-I	PD-II	PD-I vs. HC	PD-II vs. HC	PD-I vs. PD-II.
	Mean ± SD	Mean ± SD	Mean ± SD	Value of *p*	Value of *p*	Value of *p*
LGL (%foot length)	0.93 ± 0.08	0.82 ± 0.19	0.84 ± 0.20	0.142	0.268	1.000
SSL (%foot length)	0.48 ± 0.04	0.39 ± 0.11	0.40 ± 0.11	**0.008**	**0.021**	1.000
Anteroposterior position (mm)	3.87 ± 2.87	9.17 ± 4.98	5.45 ± 3.57	**0.000**	0.692	**0.018**
Lateral symmetry (mm)	2.86 ± 2.69	6.56 ± 5.54	5.63 ± 3.43	**0.045**	**0.029**	0.819

PD-I, PD baseline group; PD-II, PD cued condition group; HC, healthy control group; LGL, length of gait line; SSL, single support line. Bold values denote statistical significance at the *p* < 0.05 level.

### Relationships between spatiotemporal, kinematic and COP variables

The Table 5 shows the results of multiple linear regression analysis coefficients. All the tolerance values are more than 0.10 and the value of the variance inflation factor (VIF) are all less than 5. On the basis of these findings, we can say that there is no multilinearity among all the independent variables in the table. We can see that the value of *p* for ankle peak dorsiflexion in swing phase is less than 0.05 (*B* = −0.710, *p =* 0.000), which shows it has a negative correlation with the dependent variable, AP position. Pearson’s correlation coefficients for spatiotemporal, kinematic, and COP variables were further calculated. The relationships between AP position and ankle peak dorsiflexion in the swing phase were negatively correlated under cued condition (*r* = −0.814, *p* = 0.000) and uncued condition (*r* = −0.518, *p* = 0.028). In contrast, the Mini TC was positively correlated with ankle peak dorsiflexion in the swing phase under cued condition (*r* = 0.696, *p* = 0.001) and uncued condition (*r* = 0.525, *p* = 0.025).

**Table 5:** Result of multiple linear regression analysis coefficients.^a^

Model	Unstandardized coefficient		Standardized coefficient	*t*	Value of *p*	Collinearity statistics	
	*B*	Std. Error	Beta			Tolerance	VIF
Constant	18.302	8.892		2.058	**0.045**		
Stride length	−0.018	0.038	−0.057	−0.461	−0.647	0.610	1.640
Double stance phase	0.105	0.209	0.060	0.499	0.620	0.658	1.519
Max TC2	−25.279	27.407	0.103	−0.922	0.361	0.754	1.326
Mini TC	−42.262	55.417	0.103	−0.763	0.449	0.509	1.965
Peak dorsiflexion in swing phase	−0.710	0.146	−0.583	−4.847	**0.000**	0.647	1.546
Hip ROM in gait cycle	−0.127	0.102	−0.142	−1.254	0.216	0.728	1.374

^a^Dependent variable: Anteroposterior position; Max, maximum; Mini, minimum; TC, toe clearance; ROM, range of motion; VIF, Variance inflation factor. Bold values denote statistical significance at the *p* < 0.05 level.

## Discussion

The purpose of this study was to evaluate the immediate gait change with continuous visual cues in patients with PD. Consistent with previous research results (4, 30), the present study demonstrated marked gait improvement in spatiotemporal and kinematic measures in PD patients, under a cued condition (11). Moreover, the study proved that PD patients do not lose the ability to walk toward a normal gait, but probably experience difficulty in activating the motor control system. To our knowledge, this study is the first to address the effect of cueing on COP during walking. The change in COP variables revealed improved gait stability in PD patients during ambulation, indicating that visual cueing can trigger a more stable footstep pattern.

The PD patients, under the cued condition, displayed improved gait patterns with shortened double stance phase and a longer stride length approximated to the normal range. According to previous studies (31, 32), impaired stride length may be a factor for postural instability that possibly contributes to an increased risk of falls in patients with idiopathic PD. In PD, the deficient connection between the basal ganglia and supplementary motor area (SMA) is believed to cause impaired internal cueing within the basal ganglia, often manifesting problematic walking (33). Visual cues are proposed to bypass this deficient loop and use visual motor pathways in the lateral premotor cortex (PMC) and posterior parietal cortex (PPC), as these areas are activated through externally cued movements and paradoxical movements, respectively (34). The transverse lines maybe activate these areas regardless of surrounding environmental information (33). Thus, visual cues may aid in reactivating movement control. We expected that, compared to HC group, PD baseline group had higher cadence. However, our findings showed opposite results, which maybe related to the patients being in an “on” state or influenced by the test environment. Nevertheless, statistically significant difference was not found between the two groups. In cued condition group, PD patients focused more attention on the visual cues, perhaps resulting in a further decrease in cadence. In line with our result, Ferrarin et al. also demonstrated that PD subjects at more advanced stages (H&Y > 2) tend to be more responsive to the attentional strategy, through an increase of stride length and a compensatory decrease of cadence (35). These authors (36) thought freezing of gait was probably related to the reduction in the amplitude of movements (e.g., stride length). The effectiveness of visual cues on stride length was evident in this study, which was consistent with the research result that visual cues can improve freezing of gait (16). The prolonged double stance phase in the PD group, observed in the present study, is in line with the findings of previous research (4, 30, 32), indicating that instability is a common problem among PD patients (30). The duration of double stance phase under the cued condition was comparable to that in the HC group, suggesting that visual cues might increase gait stability in PD patients. Furthermore, reduced foot clearance when walking may increase the risk of trips and falls in people and Mini TC is the key gait cycle event for predicting tripping-falls as it occurs mid-swing during walking where forward velocity of the foot is maximum (37). Previous studies have shown that increasing toe clearance height is a main strategy used to avoid tripping (38, 39). In our study, PD patients under visual cueing condition not only obtain higher Mini TC, but also Max TC2, indicating that visual cueing may play important role in preventing falls.

The gait of PD patients was characterized by a marked reduction in movement excursion in ankle and hip joints, when compared with that in the HC group. Furthermore, the addition of visual cues enabled PD patients to achieve a larger ROM of hip joints, which was comparable to that in the HC group. Findings of previous studies (4, 30) suggest that visual cues might trigger more muscle contraction in the hip joints, and even reduce muscle stiffness which can be a manifestation of various pathologies involving the basal ganglia but the use of visual information can bypass it (40), thereby enlarging the ROM in lower limb joints and lengthening the stride length.

Additionally, the PD patients showed a significantly abnormal angle in foot strike, which was far less than that of the HC group. As PD progresses, people tend to display a flat-footed gait pattern (4, 30) and experience a high risk of falling (11, 41). However, in this study, the angle at initial contact of the ankle did not show significant improvement under the cued condition. A possible explanation for this finding is that the patients with PD were instructed to step on the laser line with their toes instead of making contact with the ground using their heels. Additionally, the ankle showed decreased dorsiflexion in swing phase at the baseline, implying that toe-floor clearance during walking was diminished in PD patients (42). Previous work has described that increased ankle dorsiflexion at midswing and also at the moment of foot strike due to an attentional strategy to strike the heel first also improved the MiniTC during walking (43). Notably, the dorsiflexion of the ankle in swing phase increased with the intervention of visual cues in present study, which may contribute to improving foot clearance. A well-accepted explanation is that light stripes placed on the travel path may be effective in helping patients to pay attention to the discrete goal, thereby unloading the emphasis on automatic motor function through alternative visual-motor circuits and allowing the well-planned execution of movement (7, 11).

Regarding the COP parameters, Shin et al. first demonstrated that the asymmetry of dynamic COP parameters was one of the characteristic features of PD patients’ gait (25). The previous study showed the decreased length of gait line (LGL), single support line (SSL), and AP position were along with increased lateral symmetry (44), which was considered a predictor of future falls (45). Our study also indicated that the abnormal trajectory of dynamic COP includes a decreased length of the gait line, both in the stance phase and single support phase, and increased asymmetry of the COP intersection point in PD patients, which was consistent with recent findings (25). In this study, we however did not observe a positive effect of visual cues on LGL, SSL, and lateral symmetry. Additionally, in this study, the AP position of COP of PD patients showed a larger value, which means more deviations of COP in the direction of progression (46), suggesting that the PD patients were in unstable conditions.

The slightly increased LGL and SSL, and reduced lateral symmetry of the COP at both sides, which exhibited no significant differences, were important gait characteristics for PD patients with visual cues. However, a large reduction in the values of the AP position was evident when PD patients were exposed to visual cues, which indicated that the PD patients became stable with the improvement in their gait performance. Moreover, the AP position was negatively correlated with the ankle dorsiflexion in swing phase. Ankle dorsiflexion weakness results in a lack of heel strike and decreased foot clearance. In present study, PD patients in cued condition presented larger ankle dorsiflexion and higher Mini TC, and a positive correlation existed between these two variables, which were consistent with previous studies (47, 48). In light from above, perhaps PD patients can achieve stable gait by increasing ankle dorsiflexion in cued condition. The reduction in AP position maybe also related to the intention of the subjects to keep their gait more stable in order to have a more stable laser projection on the floor. Similar as if they would be walking while keeping a glass of water. However, if the patients intended to keep their gait more stable in order to obtain a more stable laser projection, it was equivalent to adding an additional task, which often triggers or aggravates FOG (49). Furthermore, when visual cues were present, freezers’ gait improved regardless of the dual-task (50), which also suggests that the influence of visual cues plays a dominant role. A feedback mechanism with a real-time visual display may be used to elicit immediate gait adjustments, as it assists them to focus on stepping with normal footsteps.

Some limitations of the study should be mentioned. First, the subject numbers were relatively small and all subjects were on medication when the tests were conducted, which limits the generalizability of the results. Therefore, whether the present results can be generalized to the off-stage are unknown and a larger sample size is needed in further investigation. Second, it is not clear how long the improvement lasts and in particular if there is a “wake” effect once the device has been removed, so further studies are required to investigate the effects of cueing in the home environment, and a follow-up period to identify whether the positive effect of cue use continually remains or if more gait symmetry appears.

This study provides insights into the effect of visual cues on gait patterns and stability during walking, in patients with PD. The visual cues provided by the wearable laser device, serving as a practical substitute for ground lines, increased the toe clearance, ankle dorsiflexion in swing phase, and decreased COP deviations in AP directions. The visual cues function as external drivers that facilitate a compensatory shift toward a goal-directed mode of action control, and they probably provide an opportunity to compensate for a gait deficit, normalize the gait deviation and walking patterns. Overall, the current study suggests that visual cueing maybe an effective physiotherapy method for improving gait and stability in patients with PD. Additionally, visual cues may have a positive impact on functional mobility and quality of life. Further research is necessary to quantify long-term benefits and explore the effects of visual cues on muscle stiffness using electromyogram in PD.

## Data availability statement

The raw data supporting the conclusions of this article will be made available by the authors, without undue reservation.

## Ethics statement

The study was conducted according to the declaration of Helsinki and under the supervision of the Ethics Committee of the Affiliated Hospital of Xuzhou Medical University (Ethical approval No. XYFY2020-KL105-01). The patients/participants provided their written informed consent to participate in this study.

## Author contributions

JX, GC, and WZ contributed to the concept of the study and coordinated the study. WZ and YS analyzed the data and drafted the manuscript. YH and WS were substantially involved in the study design and data collection and made contributions to the interpretation of the data. SY initiated the study, monitored its progress, and helped to analyze the data. All authors contributed to the article and approved the submitted version.

## Funding

This work was supported by Key Research & Development Plan of China (2020YFC2006604), Key R&D Program (Social Development) of Jiangsu Province (BE2021630), the Open Funds of Jiangsu Key Laboratory of Brain Disease Bioinformation (XZSYSKF2020011), and Key R&D Program (Social Development) of Xuzhou (KC22249).

## Conflict of interest

The authors declare that the research was conducted in the absence of any commercial or financial relationships that could be construed as a potential conflict of interest.

## Publisher’s note

All claims expressed in this article are solely those of the authors and do not necessarily represent those of their affiliated organizations, or those of the publisher, the editors and the reviewers. Any product that may be evaluated in this article, or claim that may be made by its manufacturer, is not guaranteed or endorsed by the publisher.
